# Highlight Report: Hepatobiliary differentiation from human induced pluripotent stem cells

**DOI:** 10.17179/excli2020-1068

**Published:** 2020-01-23

**Authors:** Patrick Nell

**Affiliations:** 1 Leibniz Research Centre for Working Environment and Human Factors, Ardeystr. 67, 44139 Dortmund, Germany

## ⁯⁯⁯⁯⁯⁯⁯

***Dear Editor, ***

Recently, Wu and colleagues published a study about hepatobiliary organoids generated from human induced pluripotent stem cells (Wu et al., 2019[22]). For this purpose, the authors used a three-stage differentiation protocol, differentiating hiPSCs via endoderm/mesoderm to hepatoblasts and finally, hepatocyte and cholangiocyte-like cells over 45 days. They report that the resulting hepatocyte-like cells take up indocyanine green, accumulate lipid and glycogen, secrete albumin and urea, show drug metabolizing ability, and store bile acids. Moreover, the organoids survived for more than eight weeks after transplantation into immune-deficient mice (Wu et al., 2019[22]).

Several studies have used human stem cells including hiPSC and reported that differentiation via definitive endoderm resulted in hepatocyte-like features (Wang et al., 2019[21]; Cameron et al., 2015[4]; Sachinidis et al., 2019[20]; Collin de I'Hortet et al., 2019[5]; Ardalani et al., 2019[3]; Mun et al., 2019[18]). However, previous genome-wide studies comparing hiPSC derived hepatocyte-like cells to primary human hepatocytes have demonstrated that these cells have limitations with regard to their maturity and influences from other lineages (Sachinidis et al., 2019[20]; Godoy et al., 2015[10]). While a fraction of genes adopts similar expression levels as primary hepatocytes, the expression of other important gene clusters is insufficiently up- or downregulated. Moreover, unwanted genes are expressed that are not found in isolated human hepatocytes; an example is CDX2 that usually is expressed in trophoblast cells, but also colon epithelial cells and its precursors and not (or only at very low levels) in mature hepatocytes (Sachinidis et al., 2019[20]; Godoy et al., 2015[10]). 

A limitation of the present study of Wu and colleagues (2019[22]) is that it selected hepatocyte markers for demonstration of hepatocellular features, but avoids analysis of previously reported undesired features of hiPSC derived hepatocyte-like cells (Sachinidis et al., 2019[20]; Godoy et al., 2015[10]) that would indicate the state of commitment towards the hepatocyte lineage. Wu et al. (2019[22]) emphasize that they succeeded in establishing 3D organoids instead of using 2D cultures and suggest that their approach represents one step forward towards hepatic organogenesis under defined conditions *in vitro*, by allowing for mixed lineage differentiation of endoderm and mesoderm, exploiting TGF-β and Notch signaling pathways. Although the co-differentiation of biliary structures and hepatocytes shows the expression of respective markers, a systematic comparison of organoids to 2D cultures is not presented (Wu et al., 2019[22]). The use of mTeSR for stimulation of TGF-β and Notch signaling resulting in the mixed lineage configuration of the *in vitro* system was shown to lead to lower expression of genes associated with mature hepatocytes, such as albumin, while alpha-fetoprotein, which is found predominantly in immature hepatocytes, was upregulated. However, without thorough characterization on a genome-wide scale, it remains elusive if the co-differentiation has additional negative effects on hepatocyte lineage commitment and maturation. Activation of the TGF-β signaling pathway, as an example, may induce upregulation of epithelial-to-mesenchymal transition (MET) associated genes, potentially limiting hepatocyte maturation, which could be investigated by monitoring TWIST and SNAIL transcription factor activity. It should be considered that hepatocytes *in vivo* are arranged in sheets along sinusoids (Hammad et al., 2014[15]; Hoehme et al., 2010[16]; Reif et al., 2017[19]). The emergence of endothelial-like cells may be a beneficial feature with regard to transplantation and subsequent vascularization of organoids, however, they do not show organization into characteristic sinusoidal structures *in vitro*. Whether they contribute to a more successful engraftment has not been demonstrated. It would be interesting to see, whether transplantation could lead to more efficient tissue self-organization, if it occurred at an earlier time point of the co-differentiation approach. 

Currently, there is a high demand for hepatocyte *in vitro* systems for toxicity tests (Gu et al., 2018[14]; Ghallab et al., 2016[7]; Godoy et al., 2009[8], 2013[9], 2016[11]; Grinberg et al., 2014[13], 2018[12]; Ghallab, 2017[6]) and stem cell derived hepatocytes would be highly welcome (Leist et al., 2017[17]; Arbo et al. 2016[2]). Primary human hepatocytes isolated from human liver tissue still represent a gold standard (Albrecht et al., 2019[1]; Gu et al., 2018[14]). Also, the present study of Wu and colleagues (2019[22]), although presenting an interesting approach, does not yet demonstrate if their hiPSC- derived cells are truly equivalent to human hepatocytes, because a systematic genome-wide comparison to primary cells still has to be performed. 

## Conflict of interest

The author declares no conflict of interest.
